# The Hospital Saturday Fund and Lady Almoners

**Published:** 1913-02-15

**Authors:** Conrad W. Thies

**Affiliations:** Late Secretary of the Royal Free Hospital, London.


*To the Editor of* THE HOSPITAL.

### The Hospital Saturday Fund and Lady Almoners.

*To the Editor of* THE HOSPITAL.

SIR,—As a former hospital secretary with many years' experience of the method of inquiry by duly trained lady almoners which is adopted at many of our leading hospitals, I venture to make some remarks in reference to the statements contained in the letter published in your last issue from Mr. G. A. Lewcock, member of the Surgical Appliance Committee of the Hospital Saturday Fund, and the comments in your Notes thereon.

In the first place, I would desire to point out that, although all hospital managers very fully appreciate the financial assistance which they receive from the Saturday Fund, it must not be forgotten that in cases where hospital letters are given, or, as your correspondent expresses it, are *purchased*, by the Fund in return for its contribution, the cost to the hospital of the benefits conferred upon persons using such letters very greatly exceeds the amount thus contributed.

It is evident that the money paid for the letters which are issued is not by any means sufficient to defray the actual cost of the treatment which is given; and, if all the letters issued to subscribers by any hospital were fully utilised, the institution would still have to depend mainly upon the gifts of those charitably disposed supporters who do not demand any such return for their contributions. It is, I think, too often claimed that the comparatively small amounts which are subscribed by individuals through the Saturday Fund give these contributors a *right* to hospital treatment, irrespective of the suitability of such persons to receive free treatment. I do not see how it is practicable for the managers of our voluntary hospitals to ignore the consideration whether persons applying for treatment are insured persons, even if such applicants claim that they have subscribed to the Hospital Saturday Fund collections.

On the other hand, I feel sure that the managers of the hospitals are desirous of affording treatment to all cases that, in the opinion of their medical officers, are considered sufficiently serious to need the special treatment which can only be given in a hospital.

I fully realise and heartily sympathise with the difficulty which the Hospital Saturday Fund has to face owing to the provisions of the National Insurance Act, for it is clear that a very large proportion of the persons who contribute to its weekly collections are insured persons. I can only express the hope that some satisfactory solution of this difficult problem may be arrived at.

With regard to Mr. Lewcock's charge that patients are "questioned and insulted by the so-called lady almoner at certain hospitals," I can only say that from my personal observations for many years past, not only at my own, but also at various other hospitals, I have no hesitation in stating that, generally speaking, such a statement has no real foundation, and I trust that this charge will be investigated by the Saturday Fund Council. Of course, there may be isolated cases where more sympathy and tact might be shown by an individual almoner, but as a general rule these ladies have been very carefully selected and trained for the work, because they possess the requisite qualities for discharging their difficult duty with due consideration to the feelings of the patients.—Believe me, yours faithfully,

CONRAD W. THIES,
Late Secretary of the Royal Free Hospital, London.

February 8, 1913.

